# Supplementary material for: High-sensitivity pattern discovery in large, paired multiomic datasets
Source: Bioinformatics. 2022 Jun 27;38(Suppl 1):i378–85. doi: 10.1093/bioinformatics/btac232 (PMC9235493; doi:10.1093/bioinformatics/btac232)
Supplement: btac232_Supplementary_Data [file btac232_supplementary_data.zip › btac232-Supp_data/sup_clean.pdf]

Supplementary Data for

High-sensitivity pattern discovery in large, paired multi-omic datasets

Andrew R. Ghazi[1,2], Kathleen Sucipto[1], Gholamali Rahnavard[1,2], Eric A. Franzosa[1,2], Lauren J. McIver[1,2], Jason Lloyd-Price[1,2], Emma Schwager[1], George Weingart[1], Yo Sup Moon[1], Xochitl C. Morgan[3], Levi Waldron[4], Curtis Huttenhower[1,2,*]

[1]Biostatistics Department, Harvard T. H. Chan School of Public Health, Boston, Massachusetts, USA

[2]The Broad Institute of MIT and Harvard, 415 Main Street, Cambridge, Massachusetts, USA

[3]Department of Microbiology and Immunology, University of Otago, Dunedin, New Zealand

[4]City University of New York Graduate School of Public Health and Health Policy, New York City, New York, USA

*Corresponding author

E-mail: chuttenh@hsph.harvard.edu

Supplementary Methods

The details of the Hierarchical All-against-All association testing (HAllA) algorithm are explained in the following sections including data input format, filtering criteria, cluster identification, establishment of the hypothesis tree, and multiple hypothesis testing correction. Software documentation, demonstration data, and implementation details are available at https://huttenhower.sph.harvard.edu/halla.

Data preparation (general)

Input data structure. Data are paired in the form of two high-dimensional measured datasets, where the data $X_{p\times n}$ are assumed to be p predictor variables (or features) measured on n samples that give rise to d response variables contained in the data $Y_{d\times n}$. Note that column i of X is co-indexed with column i of Y, so that X and Y are aligned. Unlike previous methods of variable selection, such as partial least squares regression and PCA regression, HAllA is robust to heterogeneous data – both paired variables (i.e. the predictor and response) can be numeric, continuous, binary, or categorical. For mixed data that includes binary or categorical data, HAllA discretizes features in both datasets.

Filtering data. Datasets are filtered: only common samples (columns) are included, while features (rows) with low variability are optionally (although recommended to be) removed. Variability is defined by a maximum frequency threshold. Features whose most common value exceeds this frequency threshold (defaulting to 100%) are removed. This univariate prefilter excludes non-informative features and increases statistical power under most circumstances.

Adjusting for covariates. HAllA's hypothesis by default is for unadjusted association between the two data matrices. For adjusted association (for example, the association between microbial abundance and metabolites, while adjusting covariates such as gender, diet, etc.), the default permutation process does not generate valid null distributions. In this case, the user should provide covariate-adjusted regression residuals, instead of raw data, as the input to HAllA (Winkler, et al., 2014). This step can be involved (especially when samples have "grouping" structure, such as in a longitudinal or family design) and does require the user be familiar with the appropriate models for different types of covariates. We purposefully left it to the user's discretion so that they are aware of adjustment models used and the proper interpretation of corresponding p-values.

Data preparation (applications)

Nutrimouse dataset: fatty acid-xenobiotic metabolism associations in PPARα-deficient mice. The nutrimouse dataset is a paired dataset of 120 genes and 21 hepatic fatty acids for 40 samples. Gene expression levels have been measured using nylon microarray with radioactive labeling or genes from liver cells with potential association with

nutrition context. Hepatic fatty acid concentrations have been measured using gas chromatography.

Pouchitis datasets: the gut microbiome with host transcription in ulcerative colitis. We processed a paired dataset of 7000 microbial OTUs (relative abundances) and 19908 host transcript expressions values, drawn from 255 joint samples, as following: 1) the OTU table from the original publication was filtered to include only the 760 microbes achieving a prevalence of at least 10% for values exceeding 0.01% relative abundance. We also combined all non-further-classified "bacteria" and "unclassified" OTUs with a single "unclassified" taxon with abundance equal to the sum. 2) We used the top 10% most variant genes from the transcript data, yielding 1,991 gene expressions. We also used 688 microbes whose variability exceeded a minimal entropy of 0.5.

DiabImmune dataset: microbes with metabolites in the infant gut microbiome. In addition to the filtering of the DiabImmune microbe (20 features) and metabolite (248 features) datasets as previously published (Kostic, et al., 2015) across 104 samples, its authors applied a correction during part of its analysis to regress out temporal effects prior to associating microbes with metabolites, which we have also applied here. Briefly, a linear mixed-effects model was fit using the R lme4 package to each metabolite and OTU, of the form:

lmer(microbe ~ 1 + (1 | subject) + time, data = OTU_table)

lmer(metabolite ~ 1 + (1 | subject) + time, data = Metabolite_table)

This yields the same time-corrected residuals for HAllA analysis that were used for all-against-all comparisons in the original study.

Discretization procedure

HAllA uses an equal-frequency binning approach by default for discretizing continuously valued inputs for discretely valued similarity scores. That is, an equal number of samples are placed into each bin (with the remainder occupying the last bin). Let Z be an n-dimensional random vector. The discretization of Z, $\mathrm{disc}\,(Z)$, applies equal volume binning to Z with the number of bins equal to $\mathrm{round}(\sqrt[3]{n})$, where $\mathrm{round}(\cdot)$ indicates the integer closest to $\cdot$. However, a cut point is never chosen that would place samples with the same value in different bins. Each feature in each dataset is discretized separately but with equal numbers of bins. For example, for a continuous feature with 95 samples there would be 5 bins in each feature, each containing 19 samples. For a dichotomous feature with 95 samples, two bins will result because there are only two possible values.

Hierarchical testing of block-structured hypotheses

The dependency of performed tests has been discussed as a great challenge when accounting for multiple tests with FDR correction (Xie, et al., 2011). Accounting for correlated tests has been widely studied in other areas such as variable selection in

genome-wide association studies (GWAS) and other high-throughput data analysis to reduce the data dimension (Dehman, et al., 2015).

We form a hypotheses tree that structures tests of association hierarchically by pairing clusters in increasingly specific levels of a pair of data hierarchies (which are themselves formed by hierarchically clustering a pair of input feature by sample datasets). The root of the hypothesis tree is a hypothesis node corresponding to a test of global association between the two datasets. This node is not evaluated. Descendant hypothesis nodes correspond to specific tests of association between clusters of related features in the two input datasets. The mechanisms of identifying homogeneous clusters for association testing and their insertion into the hypothesis tree are detailed below.

Hierarchical clustering. Given a similarity measure $s(\cdot,\cdot)$, average-linkage clustering is used to build a hierarchical clustering tree on each dataset: T_X (from X) and T_Y (from Y).

Descending in sub-hypotheses of block hypotheses. Clusters formed from each first cut of two datasets hierarchies are paired in hypothesis tests for association to create a single level of a hypothesis tree. Each node in the hypothesis tree represents a null hypothesis comparing a cluster in X and a cluster in Y. The root of the hypothesis tree (level 0) corresponds to a test of association between the entirely of T_X and the entirety of T_Y, but is not evaluated. In level 1, we pair all subclusters from the first cut of T_X and the first cut of T_Y. Paired clusters form a block hypothesis, and if the pairwise associations under a block pass a defined threshold for false negative tolerance (FNT) we report it as a block association. Otherwise, we descend one level through each dataset's hierarchy and examine the next prospective cuts to the trees. We calculate the Gini score improvement from each of the two prospective cuts and choose the one leading to a higher improvement to generate the next two block hypotheses. If the difference between the two Gini score improvements is close to zero (according to a user-configurable parameter, the "Gini uncertainty level", defaulting to 0.02) and the hypothesis block in question has more than 25 elements, we cut the tree that contains more nodes (the longer axis of the block). The resulting new subclusters for a block hypothesis are paired to establish new sub-hypotheses. We repeat this procedure for each node until the FNT condition is satisfied or we reach to blocks of singleton clusters. A block structured hypothesis pass FNT threshold that the fraction of FDR passed pairwise tests within it is more than a target FNT threshold.

Gini improvement uncertainty. The default Gini uncertainty level was chosen by simulating hypothetical cuts of 100x100 hypothesis blocks with 5% true associations but no true block structure and observing that 50% of simulations usually showed a difference of 0 +/- 0.02. Additional simulations that varied axis length from 3 to 560, true association fractions from .01 to .5, and cut proportions from .01 to .5 showed that only about 20% of the most extreme simulations had uncertainty intervals that exceeded this threshold.

The following pseudo-code defines our cutting and pairing approach to report block associations with FNT threshold (**Algorithm 1**):

**Algorithm 1**	*BlockAssociations*(T_X, T_Y)
**input**	T_X, T_Y *are root nodes of two binary trees from the hierarchical clustering of features*

```
H ← hypothesis(T_X, T_Y) # creates a hypothesis node
pairClustersToHypotheses(H)
return H

pairClustersToHypotheses(h, Gini_uncertainty_level):
    if not h.x.isTip() or not h.x.isTip():
        C_X ← cutBranch(h.x) #function is described in text
        C_Y ← cutBranch(h.y)
        Gini_diff ← Gini_score(C_X) - Gini_score(C_Y)
        if abs(Gini_diff) < Gini_uncertainty_level:
            if h.x.get_count() > h.y.get_count():
                C_Y ← h.y # don't cut the Y (left) hierarchy
            else:
                C_X ← h.x # don't cut the X (right) hierarchy
        else if Gini_diff > 0:
            C_Y ← h.y
        else:
            C_X ← h.x
        for c_x in C_X:
            for c_y in C_Y:
                h_child ← hypothesis(c_x, c_y)
                if FNT(h_child):
                    report(h_child)
                else:
                    pairClustersToHypotheses(h_child, Gini_uncertainty_level)
```

**output**	*Report significant associations*

Obtaining a *p*-value. We have implemented two approaches for calculating *p*-values: 1) the method described by Knijnenburg et al. (Knijnenburg, et al., 2009) to estimate *p*-value by fitting a model using generalized Pareto distribution (GPD) on the tail of null samples for the permutation test, and 2) a standard permutation test using the empirical cumulative distribution function (ECDF). The *p*-value is this case is calculated as a number of similarity score of permutated representative vectors that are greater than similarity score of original representative vectors divided by total number of permutations, plus the pseudo count of 1/*N*. This process is described in **Algorithm 2.**

**Algorithm 2**	***permutationTest*** (x, y, M, speedup, alpha)
**input**	x is a feature from X dataset<br>y is a feature from Y datasest<br>M *is the number of permutations to perform*<br>speedup is a Boolean indicating to optionally cut short permutation iterations<br>alpha is a significance-level target used when cutting short permutations

```
permutationTest(x, y, M):
  s_0 ← similarity(x, y)
```

```
s_null ← []
for i in 1 to M:
   y_p ← permute(y)
   s_null[i] ← similarity(x, y_p)
   if (i+1) % 100 = 0 and speedup:
      current = (sum(s_null ≥ s_0) + 1) / (i + 1)
      if current > alpha and (i+1) ≥ 300:
         break
if count(s_null ≥ s_0) ≥ 10:
      p_value ← (sum(s_null ≥ s_0) + 1) / (M + 1)
else:
   # use generalized Pareto distribution fit to the tail of the sampled null distribution
   # to estimate the p-value
   p_value ← estimate_pvalue_gpd(s_null, s_0)
return p_value
```

**output**	*p*-value

Similarity measures: HAllA's implementation and hypothesis testing scheme are general, allowing them to be used with a variety of similarity measures. The recommended similarity measures are: 1) Spearman coefficient (default for HAllA) for continuous data, and 2) normalized mutual information (NMI) for mixed data (continuous, categorical, and binary data). Other similarity measures implemented in the current version of HAllA include: Spearman correlation, discretized normalized mutual information, Pearson correlation, xicor (Chatterjee, 2020), and distance correlation (dCor).

The Benjamini–Hochberg (BH) procedure (Benjamini and Hochberg, 1995) is used on all hypotheses for a given level for false discovery rate (FDR) control.

Definition (Level of p-values). Consider level l of T_H. Let all the p-values (from tested and untested nodes) be denoted by P_l. Then the level l of p-values is P_l.

Definition (Performed test). Consider a hypothesis node, $H(c_x, c_y)$, with corresponding p-value p_{xy}. The hypothesis node has had a performed test if p_{xy} is derived from a permutation test comparing c_x and c_y.

Comparison with Yekutieli's hierarchical procedure: HAllA's approach was inspired by the method of Yekutieli (Yekutieli, 2008), but HAllA is designed to use a hierarchy of increasingly specific hypotheses in a distinct (and opposite) way. HAllA descends into more specific hypotheses of a parent hypothesis *H* when it fails to reject the null hypothesis for *H*. While this implies that the two blocks of features tested for association in *H* are not globally related, more specific subclusters of those blocks may yet be related, and so HAllA descends into *H* to evaluate those more specific hypotheses. Yekutieli takes the opposite approach: descending when the null hypothesis is rejected, and halting when we fail to reject the null hypothesis. This is appropriate in a testing framework where more specific follow-up tests are warranted by a highly sensitive parent test. For example, a significant ANOVA test between a categorical and continuous variable might warrant

follow-up comparisons of the individual categorical levels (i.e. post hoc t-tests), while such testing would be unwarranted if the parent ANOVA test failed to achieve significance.

Evaluation

Evaluation involved generating synthetic data with known cluster associations and subsequently assessing HAllA's performance across different tree descent methods, cluster decomposition methods, cluster linkage methods, data discretization methods, and pairwise similarity measures.

Synthetic data generation. Each simulated dataset pair consists of two datasets, $X_{p\times n}$ and $Y_{d\times n}$, for evaluation along with a true association matrix: $A_{p\times d}^{true} = \left[a_{ij}^{true}\right]$, where a_{ij}^{true} is an indicator of whether feature i in X is associated with feature j in dataset Y. Each dataset consists of n samples drawn from a multivariate normal or uniform distribution with block diagonal covariance structure. Associations between clusters were generated by associating each feature of a cluster in $X_{p\times n}$ with all features in a cluster in $Y_{d\times n}$. The structure of paired datasets was defined by:

- **Association type:** the linkage relationship between clusters in the paired datasets. Implemented options include parabola, line, log, step, L-shape, and sinusoidal (default parabola).
- **Distribution type:** the distribution of values within a feature, normal or uniform (default uniform).
- **Number of features:** the number of features within each dataset (default 500).
- **Number of samples:** the number of samples within each dataset (default 50; hence the feature/sample ratio is high, consistent with "high-dimensional" data).
- **Number of blocks:** the number of non-singleton clusters within each dataset (default 13).
- **Cluster fraction:** the percentage of features that should be part of a non-singleton cluster (default 1.0).
- **Association fraction:** fraction of the tests that they are true associations.
- **Within noise:** a parameter used to influence the degree of dissimilarity between features within a cluster (default 0.25).
- **Between noise:** a parameter used to influence the degree of dissimilarity between associated clusters (default 0.15).
- **Structure type:** the overall structure of clusters in the two datasets. It can be "balanced" (clusters have similar numbers of features; the default) or "imbalanced" (clusters have a variety of sizes).

In **Supplementary Table 4**, we have listed the synthetic data properties that we have generated for evaluation in **Fig. 2**.

Association type	Distribution type	Number of features	Number of samples	Number of blocks	Association fraction	Within noise	Between noise	Structure type
**Parabola**	Uniform	200	50	10	0.05	0.15	0.15	Balanced
**Line**	Uniform	200	50	10	0.05	0.4	0.35	Balanced
**Step**	Uniform	200	50	10	0.05	0.25	0.25	Balanced
**Categorical**	Uniform	200	50	10	0.05	0.2	0.2	Balanced
**Sine**	Uniform	200	50	10	0.05	0.4	0.3	Balanced
**Log**	Uniform	200	50	10	0.05	0.3	0.15	Balanced
**Mixed**	Uniform	200	50	10	0.05	0.25	0.25	Balanced

Supplementary Table 4: List of properties of synthetic data used by HAllA for evaluation. “halladata” is script that we designed to generate synthetic data. Here we show a set of properties the synthetic data that has been used for evaluation and also a list of parameters used by “halladata” to generate them.

We implemented a synthetic data generator (halladata) to produce the paired datasets used in our evaluation based on the above parameters. We have packaged this method as a utility script within HAllA to aid users wishing to reproduce our evaluations or to evaluate alternative approaches.

Power and FDR with varying input and block structure

Using a similar simulation-based approach, we assessed HAllA’s power when varying sample size, the number of true block associations, and the number of features in the input datasets. We evaluated over a grid of parameters as given in the table below, using linear associations, then ran 50 iterations of HAllA and AllA with the Pearson association metric to compare their power and FDR. A small subset of grid points were discarded because of limitations in the data simulating function. We used higher within-group noise (0.55) compared to Figure 3 in order to show a larger range of power values. The results are shown in Supplementary Figures S6 and S7.

Parameter	Values
Sample size	50, 100
Number of blocks	3, 6, 10, 20
Number of X features	10, 20, 50, 100
Number of Y features	10, 20, 50, 100

Analytic Power of detecting a block during tree descent

While the generality and adaptiveness of HAllA make it difficult to assess the performance of HAllA analytically, with a set of overly-conservative assumptions it is possible to exactly derive HAllA's power to successfully identify a dense block of associations during the descent process. If 1) a block of size A contains all true associations derived from relationships obeying the standard linear regression normality assumptions with a specified effect size and 2) the global all-by-all matrix of size M uses Bonferroni corrected p-values to call significant associations, and 3) we assume within-dataset correlation of features only increases the probability of detecting marginal associations, we can model the lower bound of the probability of successfully identifying a dense block with a binomial distribution.

P(accept block) = P(block density > 1 - FNT)

block density ~ Binom(A, β) / A

Here β represents the probability of successfully identifying a linear relationship with a specified effect size using Pearson correlation as the association metric, which is in essence the standard F-test underlying linear regression. By assuming that the marginal associations are independent draws using the same probability of detection β, we are ignoring the within-dataset correlation dependence that realistically should *increase* the chance of marginal association. This implies that the function below gives the lower bound of power of detecting the block. The mathematical expression for this is quite large, but the corresponding R code (partially adapted from the source code of pwr::pwr.f2.test) shown below is not.

```
block_power_bound = function(block_size, total_size, effect_size, n, fnt) {

  # only one explanatory feature in pairwise associations
  u = 1

  # Remaining degrees of freedom go to the second parameter
  v = n - 2

  lambda = effect_size * (u + v + 1)

  sig.level = .05 / total_size # Bonferroni corrected p-value threshold

  beta = pf(qf(sig.level, u, v, lower = FALSE),
            u, v, lambda, lower = FALSE)

  1 - pbinom((1-fnt) * block_size,
             size = block_size,
             prob = beta)
}
```

Note that the effect size is encoded according to the relationship to R^2 by the following equation: effect_size = R^2 / (1-R^2). Figure S8 shows an example of how the power lower bound changes as a function of effect size with a selection of other parameters.

Further note that this only applies to the situation where the HAllA descent algorithm is directly considering the proposal block in question.

Supplementary Figures

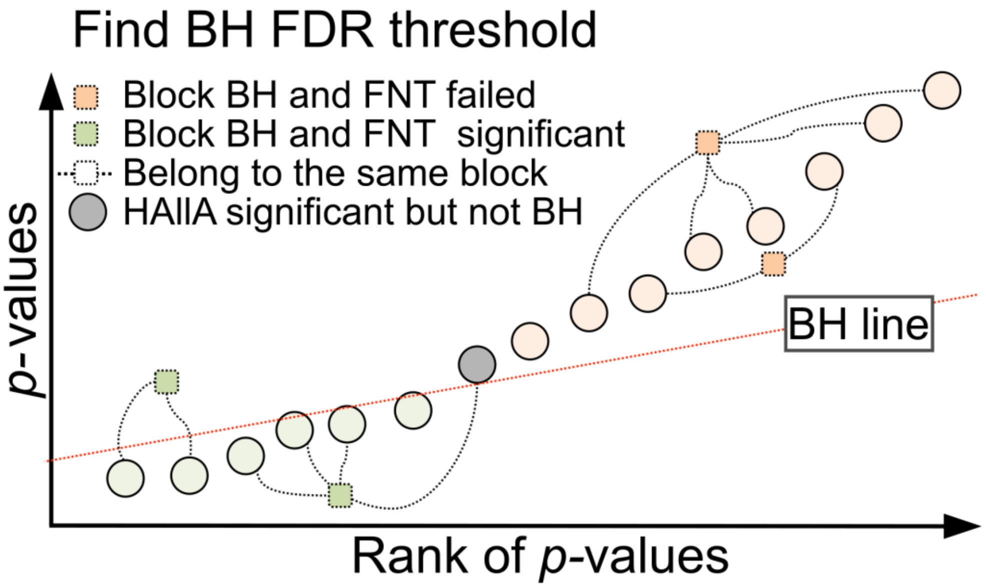

Supplementary Figure S1. HAllA improves the power of marginal all-against-all association. Each dot represents the association of an individual pair of features. The grey dot, which would typically not get called as a significant association because it falls above the BH line representing a particular FDR threshold, is captured by HAllA because its block is otherwise densely associated with related feature pairs.

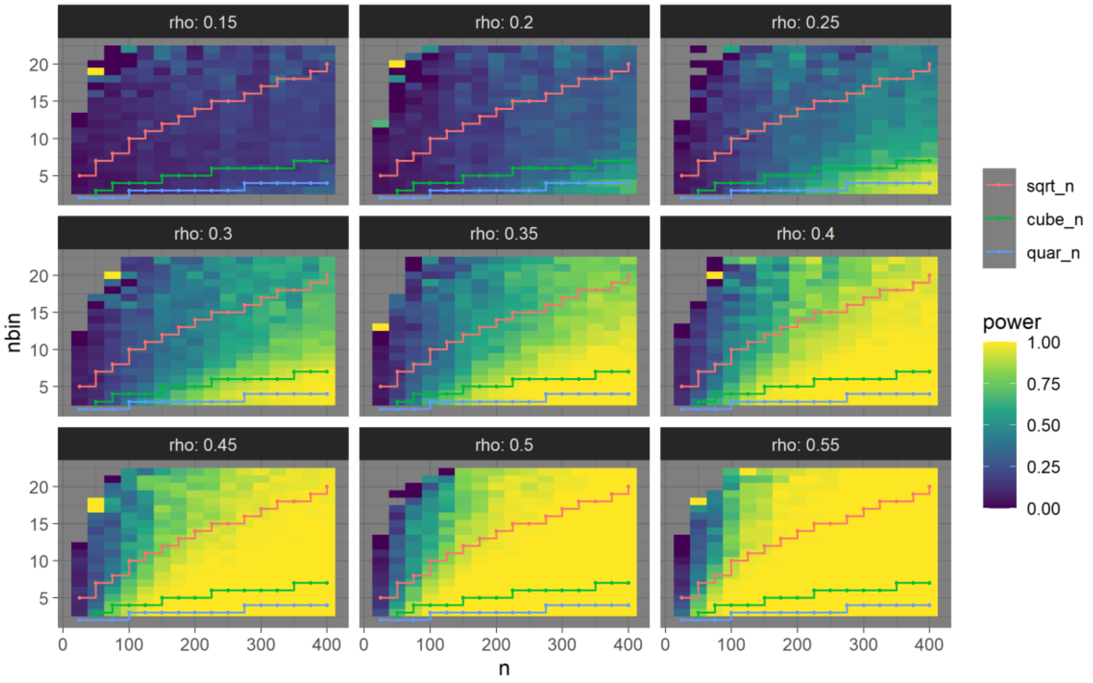

Supplementary Figure S2. Discretization into cube root n bins provides reasonable power for association discovery by mutual information. To determine an effective strategy for deciding on the number of bins for discretization, we ran a simulation that generated synthetic data with a predetermined Pearson correlation (rho) and varying sample size. We discretized this data into either the square root, cube root, or quartic root of the sample size n, with n varying from 25 to 400. We then assessed the empirical power of discovering an association by mutual information at $p < 0.05$ for 200 simulation iterations. The plot above shows the power surface as a function of sample size (horizontal axis), the number of bins, (vertical axis) and rho (color). Based on this, we selected cube root n as the default number of bins for providing reasonable power without excessive information loss.

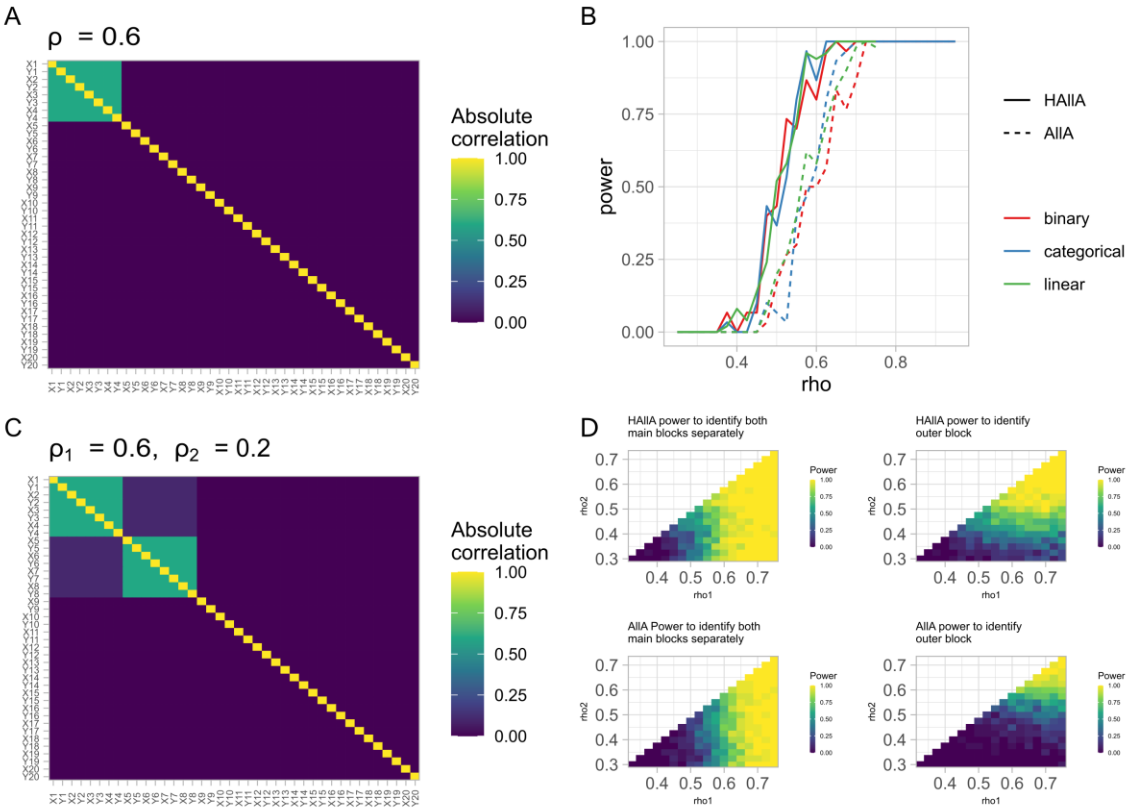

Supplementary Figure S3. Power characteristics of HAllA with varying association strength. We used simulated data with varying association strength and structure to examine the power characteristics of HAllA. A) A heat map showing the example correlation matrix used to generate simulated data with a single block of 8 features (X1-X4 and Y1-Y4) with pairwise associations with a Pearson correlation of 0.6 After drawing samples with this association structure, alternating columns in the output were assigned to two simulated datasets. B) The power curve showing HAllA's ability to successfully detect the X1-X4;Y1-Y4 association block as a function of the correlation parameter rho for binary ($n = 50$), linear ($n = 50$), and categorical ($n = 100$, 5 equi-probable categories) simulated data C) A correlation matrix heat map similar to A, showing two strong association blocks (with correlation rho1) nested within a weaker association block (with correlation rho2, where rho2 < rho1) D) The power surfaces to detect both of the strong blocks shown in C and to detect the outer block. Notably the first sub panel has a vertical boundary, while the second subpanel has a boundary that is slightly angled, showing that higher rho1 can accommodate a lower rho2 and make it easier to detect the larger, weaker outer block.

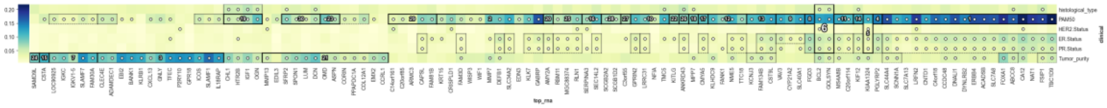

Supplementary Figure S4. HAllA reveals associations between phenotypes (mixed clinical data) and microbial species abundances in the human gut. For 526 samples from the TCGA-BRCA cohort, we tested for associations between 179 highly abundant and variable (mean expression and expression standard deviation both exceeding the 90th percentile) RNA transcripts and 19 clinical variables: years to birth, tumor purity, pathologic stage, pathology T, M, and N stages, histological type, number of lymph nodes, PAM50 subtype, ER Status, PR Status, HER2 Status, gender, radiation therapy, race, ethnicity, median overall survival, overall survival, and status. A target FDR of 0.05 and FNT of 0.1 was used with NMI as the similarity measurement.

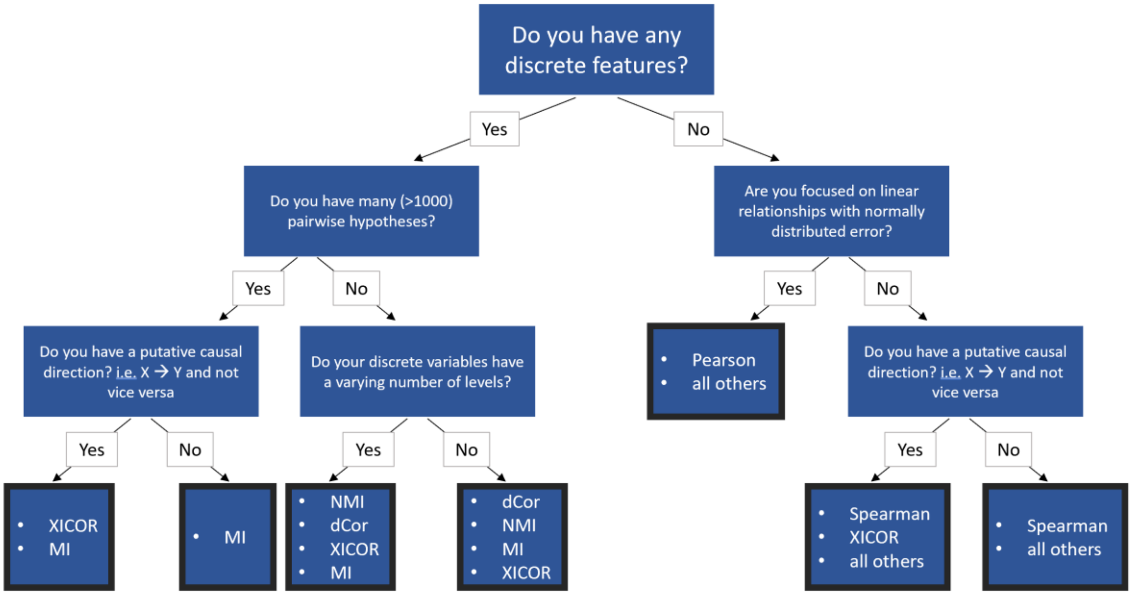

Supplementary Figure S5: Association metric guidance flowchart. This flowchart provides guidance on the selection of association metric to use with HAllA. The final box of each branch lists the association metrics that could be appropriate in decreasing order of typical effectiveness.

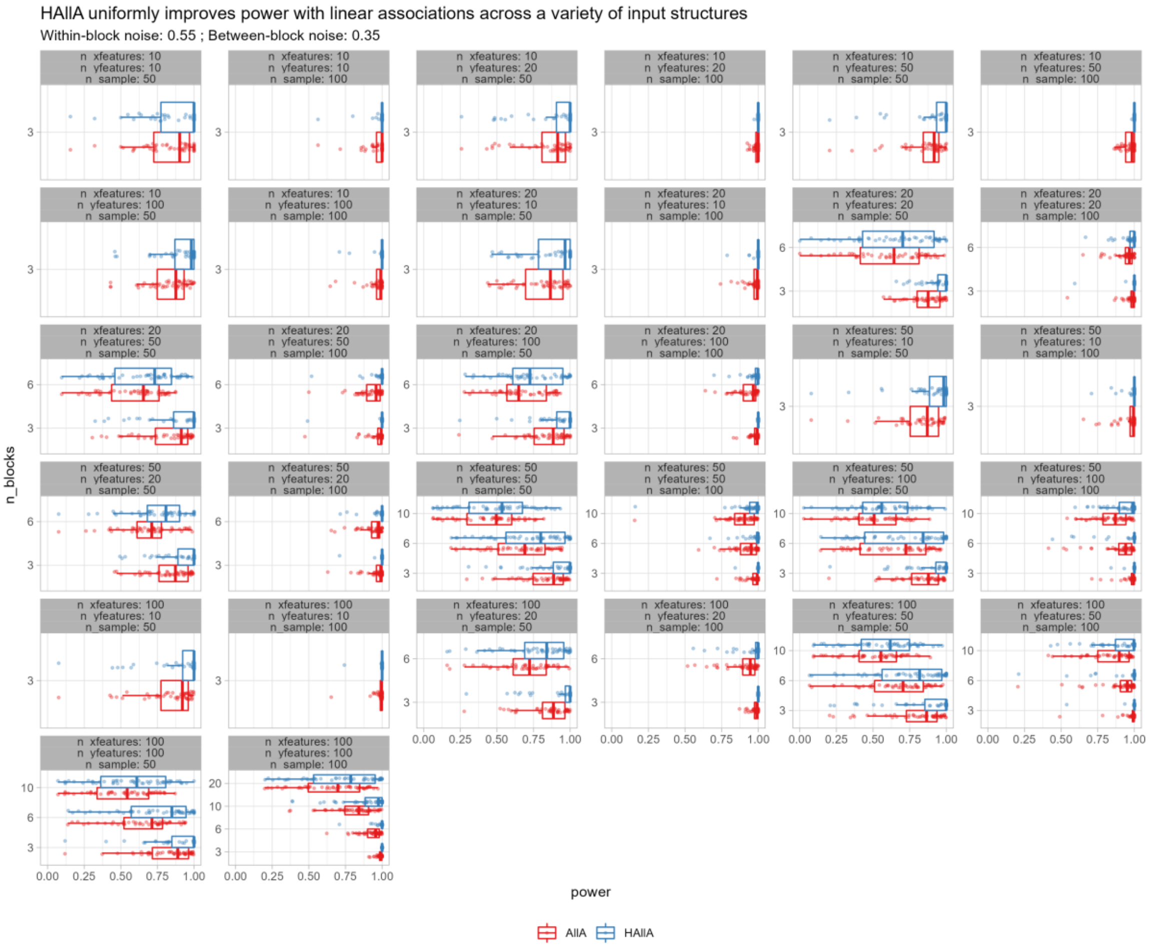

Supplementary Figure S6: HAllA power distribution over simulations with varying sample size, block number, and input dimensionality.

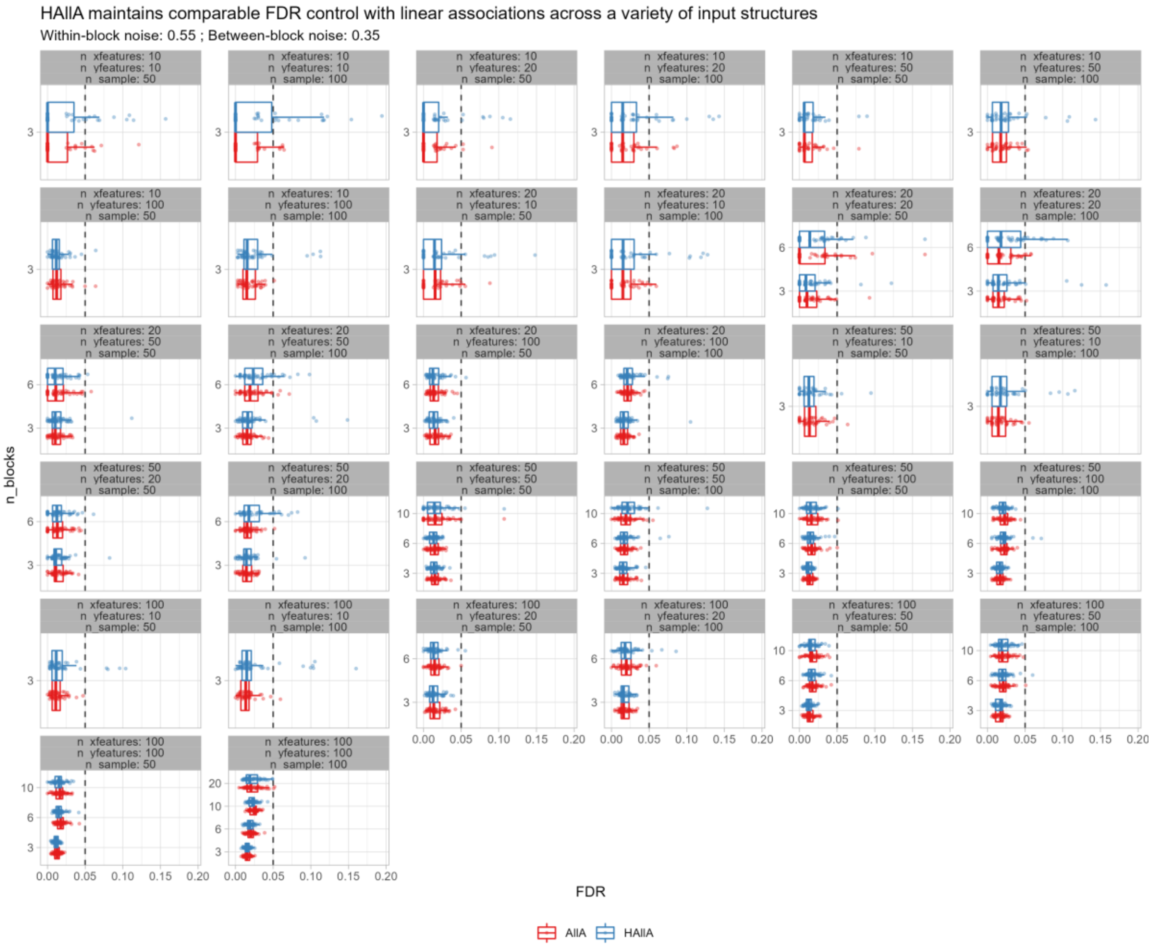

Supplementary Figure S7: HAllA FDR distribution over simulations with varying sample size, block number, and input dimensionality.

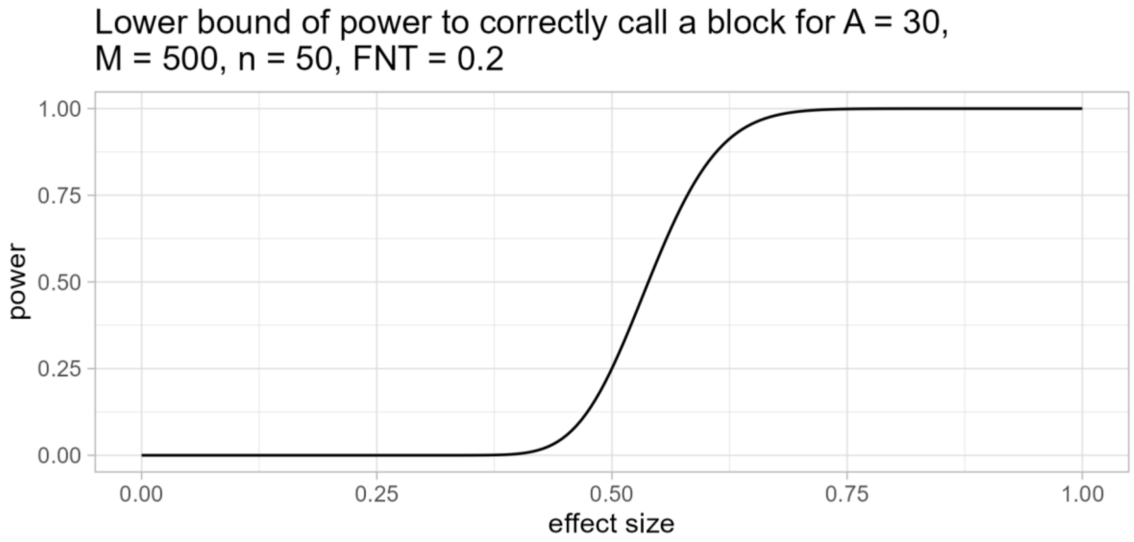

Supplementary Figure S8: Analytic power of detecting a proposal block during the descent process. By modelling the presence of marginally significant associations in a proposal block as a binomial draw, we can analytically derive the lower bound of probability of accepting the block. Assuming linear relationships and Bonferroni corrected p-value thresholds for significance, a block of size A = 30 from a global all-by-all matrix of size M = 500 will be successfully identified as a dense block according to this power curve. Note that the effect size is encoded according to the relationship to R^2 by the following equation: effect_size = R^2 / (1-R^2).

Supplementary References

1. Benjamini, Y. and Hochberg, Y. Controlling the false discovery rate: a practical and powerful approach to multiple testing. *Journal of the Royal Statistical Society. Series B (Methodological)* 1995:289-300.
2. Stephane Champely (2020). pwr: Basic Functions for Power Analysis. R package version 1.3-0. https://CRAN.R-project.org/package=pwr
3. Chatterjee, S. A New Coefficient of Correlation. *Journal of the American Statistical Association*. 2020:1-39.
4. Dehman A, Ambroise C, Neuvial P. Performance of a blockwise approach in variable selection using linkage disequilibrium information. BMC bioinformatics. 2015 Dec;16(1):1-4.
5. Han, J., Pei, J. and Kamber, M. Data mining: concepts and techniques. Elsevier; 2011.
6. Knijnenburg, T.A.*, et al.* Fewer permutations, more accurate P-values. *Bioinformatics* 2009;25(12):i161-i168.
7. Kostic, A.D.*, et al.* The dynamics of the human infant gut microbiome in development and in progression toward type 1 diabetes. *Cell host & microbe* 2015;17(2):260-273.
8. Martin, P.G.*, et al.* Novel aspects of PPARα-mediated regulation of lipid and xenobiotic metabolism revealed through a nutrigenomic study. *Hepatology* 2007;45(3):767-777.
9. Morgan, X.C.*, et al.* Dysfunction of the intestinal microbiome in inflammatory bowel disease and treatment. *Genome biology* 2012;13(9):R79.
10. Rousseeuw, P.J. Silhouettes: a graphical aid to the interpretation and validation of cluster analysis. *Journal of computational and applied mathematics* 1987;20:53-65.
11. Winkler, A.M.*, et al.* Permutation inference for the general linear model. *Neuroimage* 2014;92:381-397 %@ 1053-8119.
12. Xie J, Cai TT, Maris J, Li H. Optimal false discovery rate control for dependent data. Statistics and its interface. 2011;4(4):417.
13. Yekutieli, D. Hierarchical false discovery rate–controlling methodology. *Journal of the American Statistical Association* 2008;103(481):309-316.
