# Supplementary figures and images for: High-sensitivity pattern discovery in large, paired multiomic datasets
Source: Bioinformatics. 2022 Jun 27;38(Suppl 1):i378–85. doi: 10.1093/bioinformatics/btac232 (PMC9235493; doi:10.1093/bioinformatics/btac232)

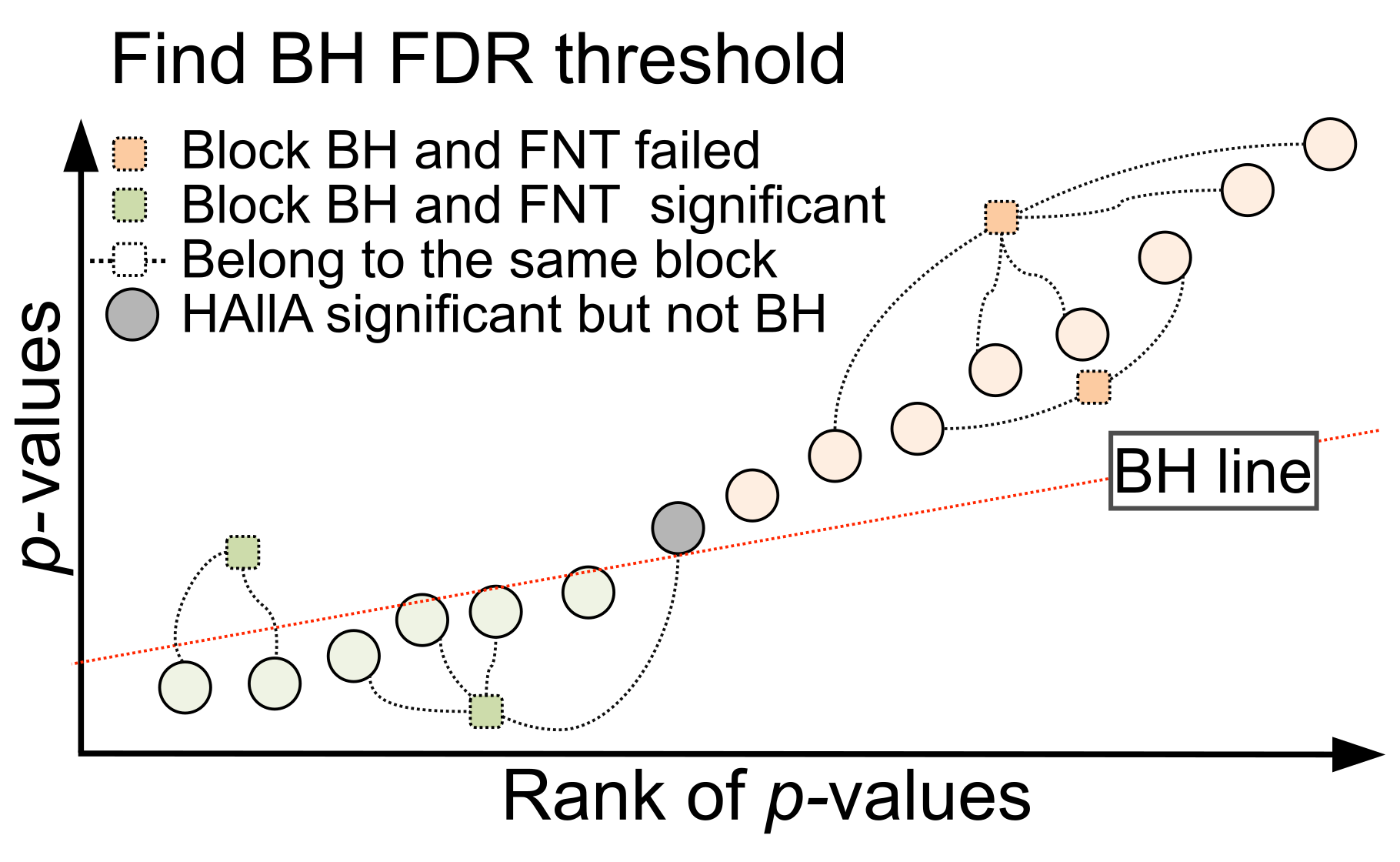

Supplement: btac232_Supplementary_Data [file btac232_supplementary_data.zip › btac232-Supp_data/s1.png]

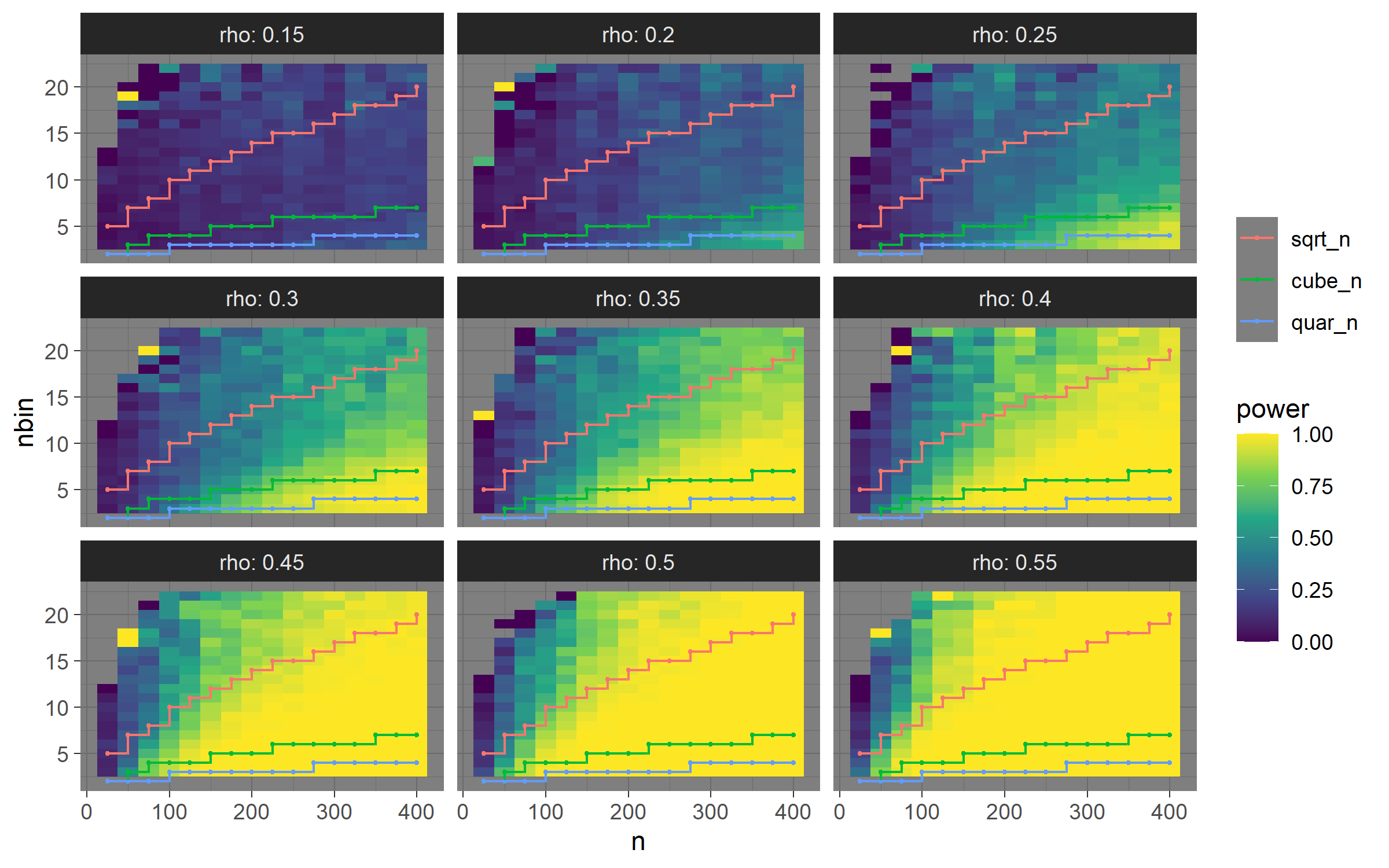

Supplement: btac232_Supplementary_Data [file btac232_supplementary_data.zip › btac232-Supp_data/s2.png]

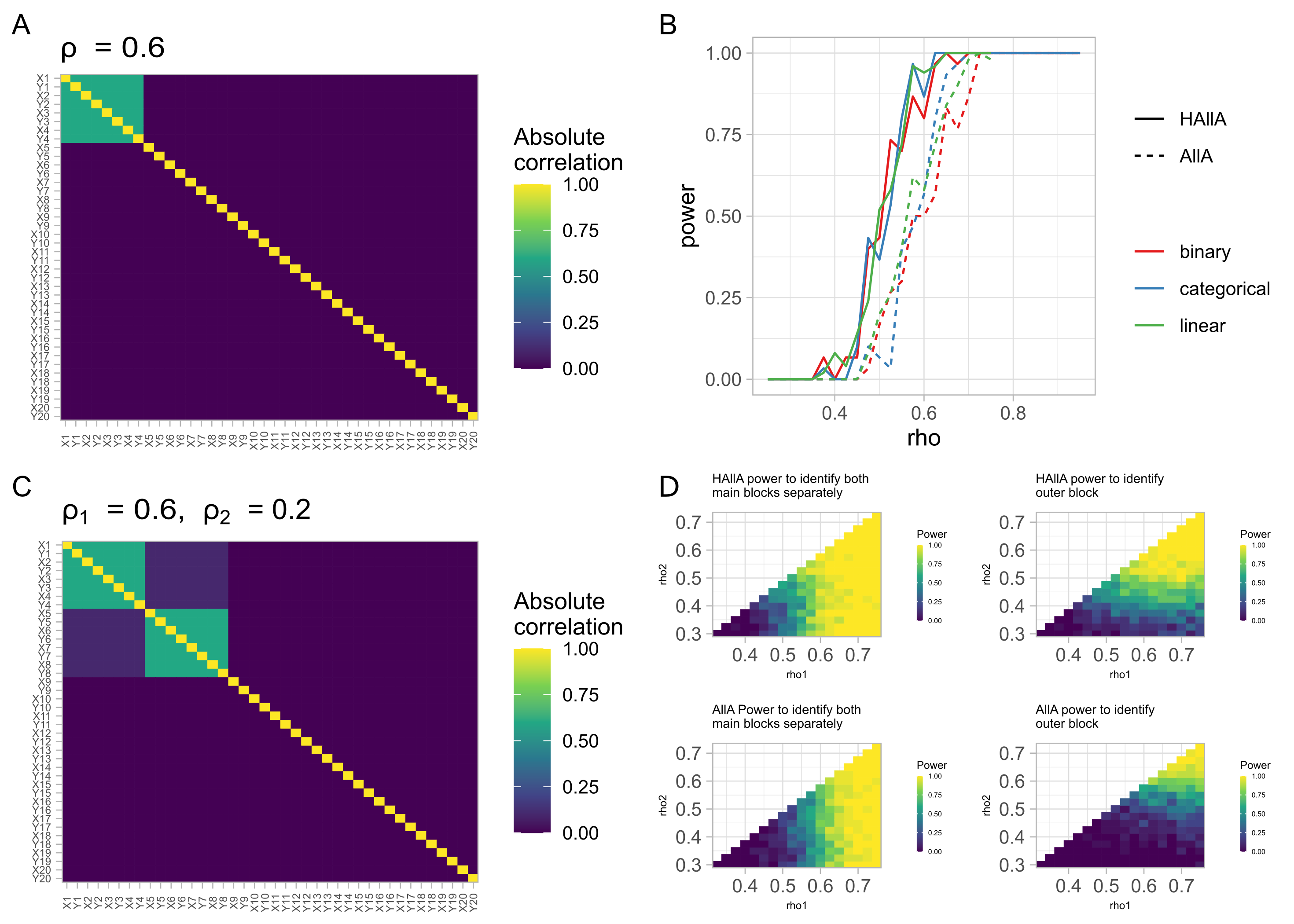

Supplement: btac232_Supplementary_Data [file btac232_supplementary_data.zip › btac232-Supp_data/s3.png]

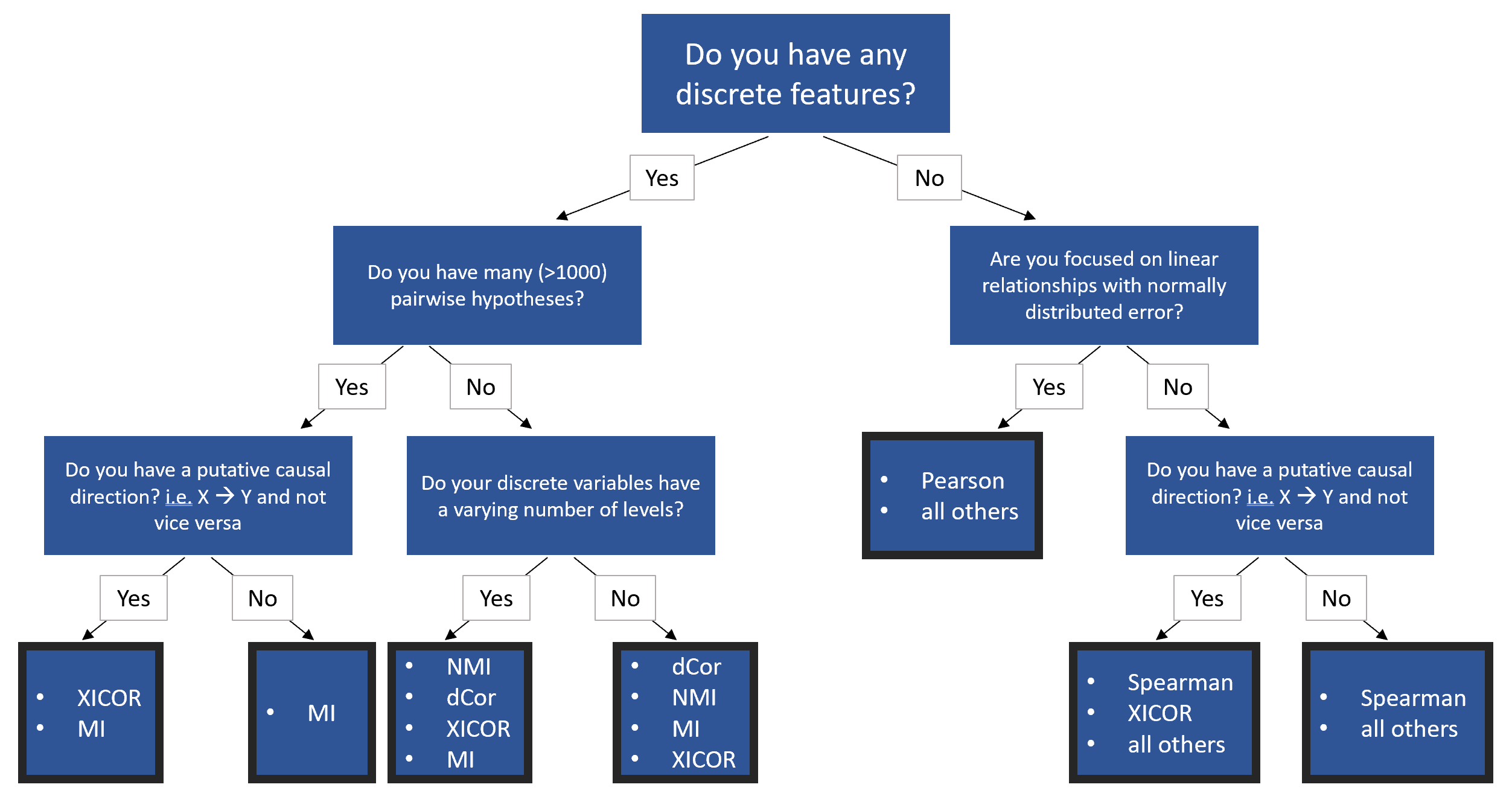

Supplement: btac232_Supplementary_Data [file btac232_supplementary_data.zip › btac232-Supp_data/s5.PNG]

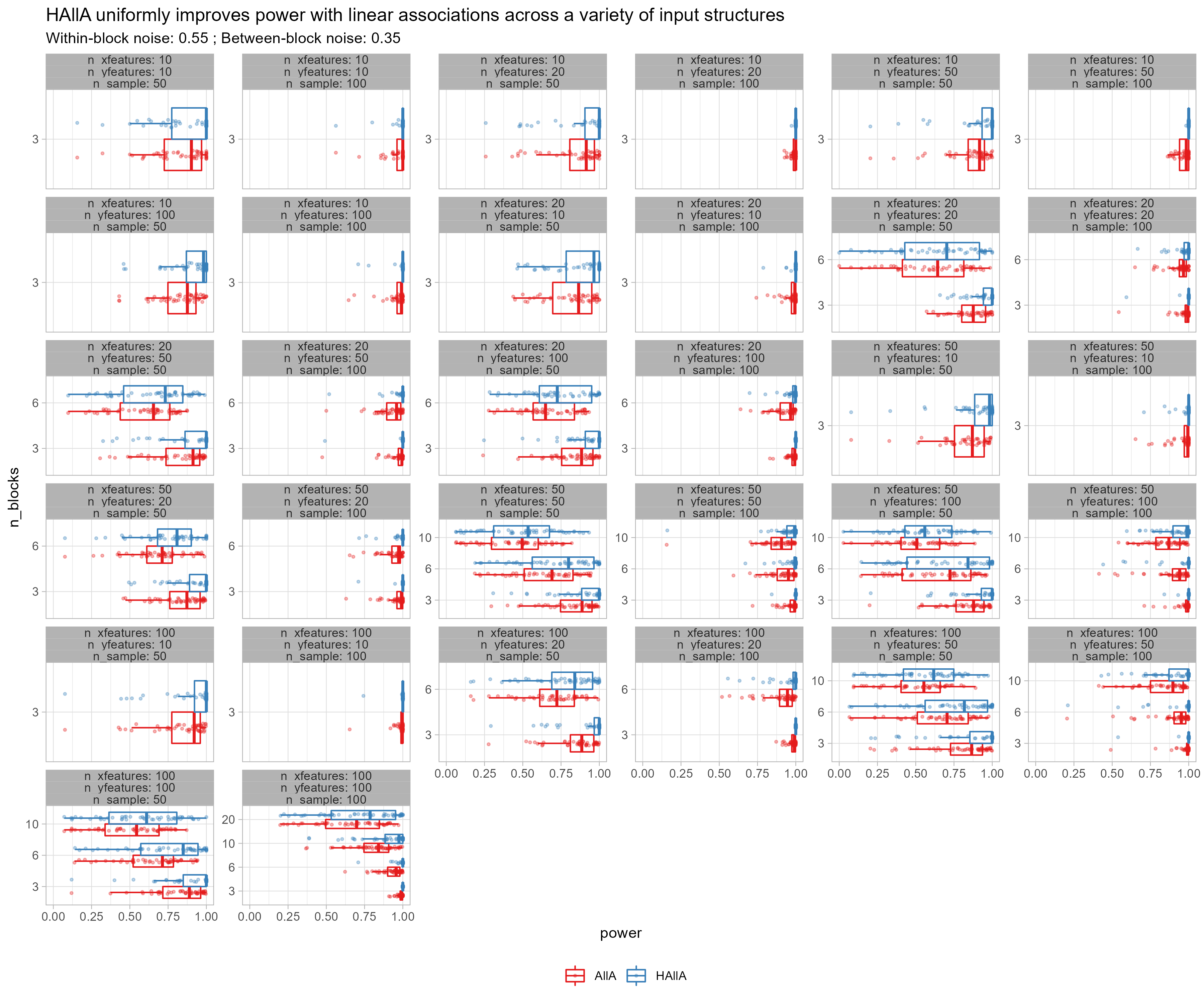

Supplement: btac232_Supplementary_Data [file btac232_supplementary_data.zip › btac232-Supp_data/s6.png]

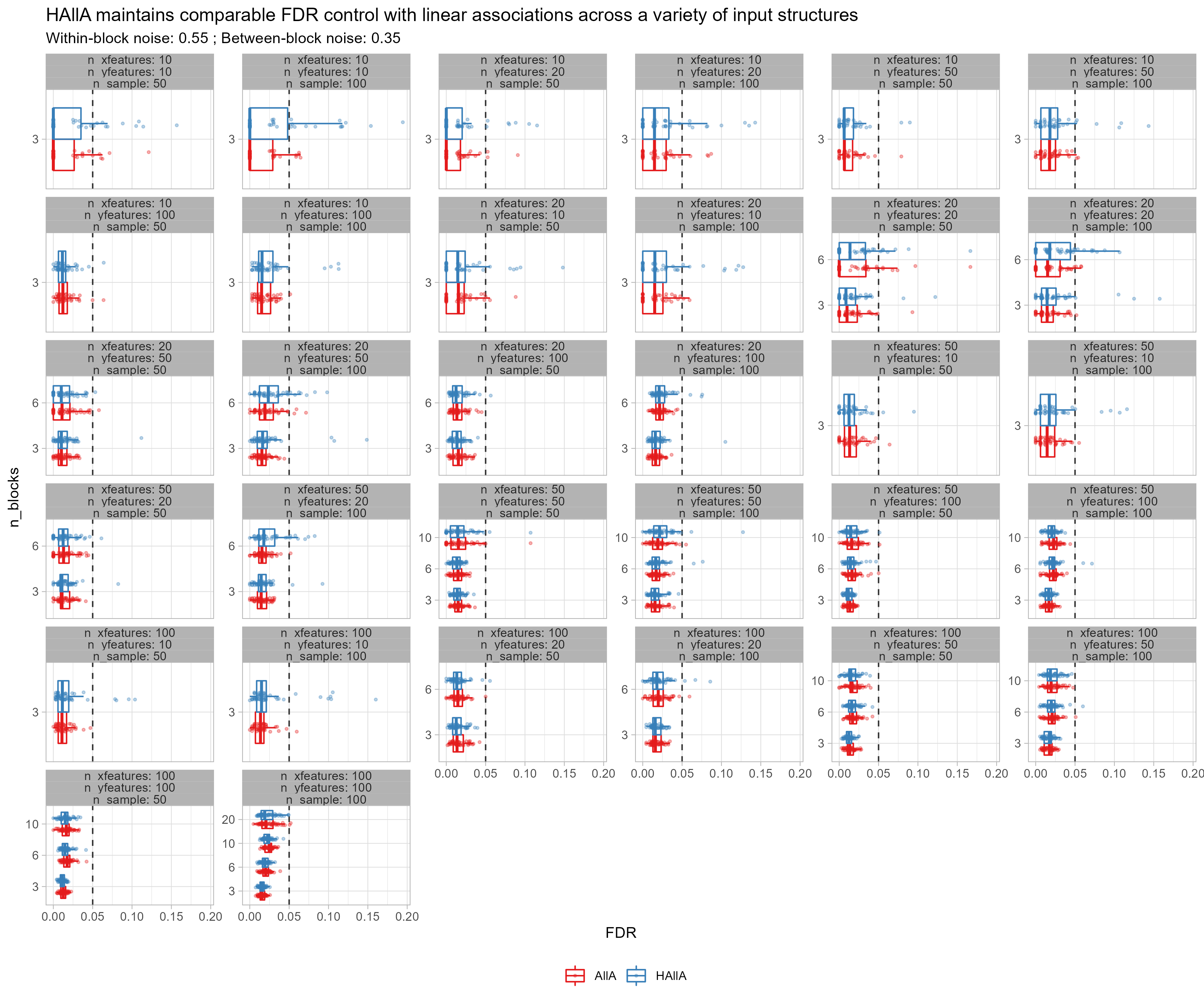

Supplement: btac232_Supplementary_Data [file btac232_supplementary_data.zip › btac232-Supp_data/s7.png]

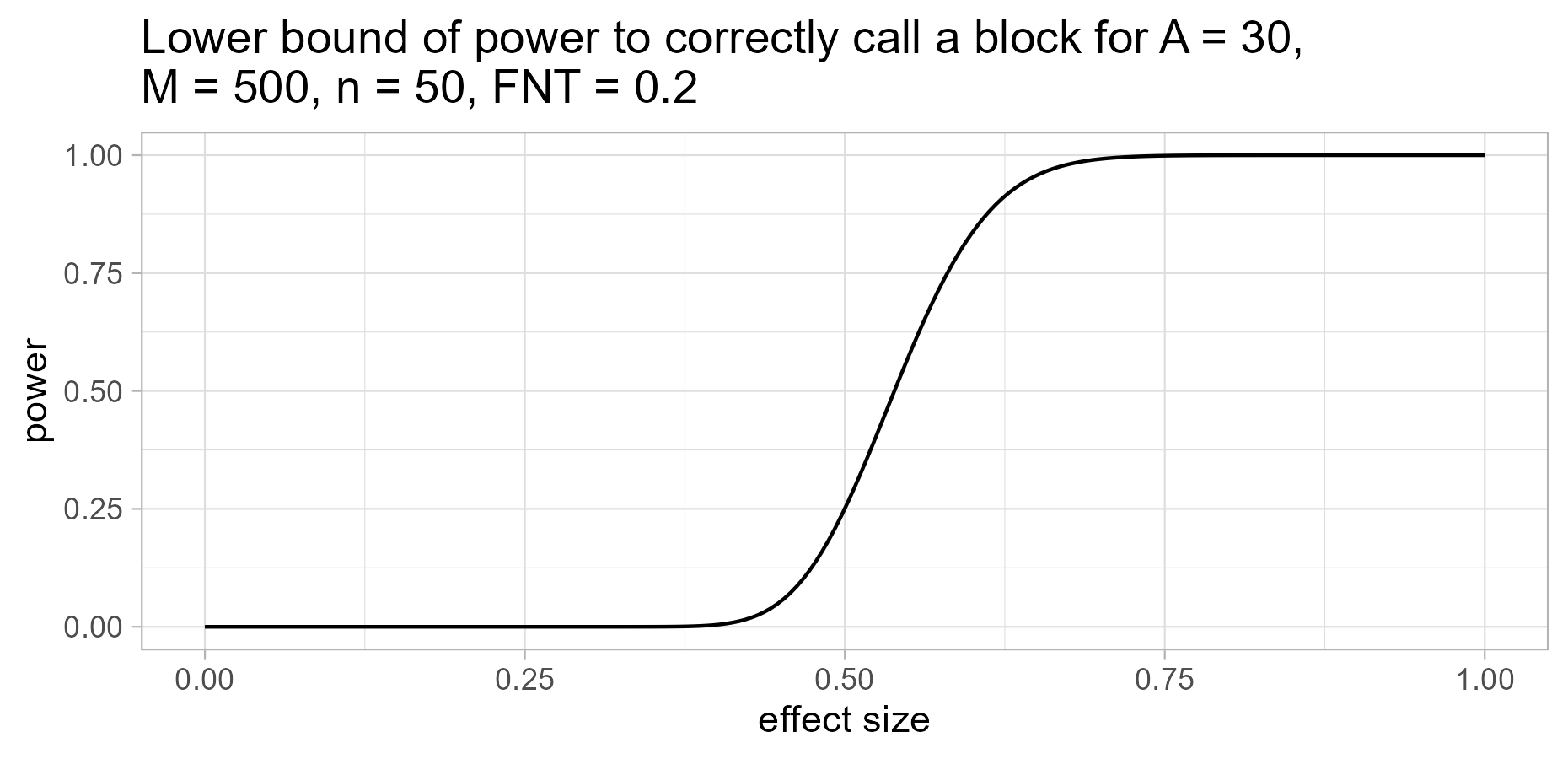

Supplement: btac232_Supplementary_Data [file btac232_supplementary_data.zip › btac232-Supp_data/s8.png]
